# The Blood of Healthy Individuals Exhibits CD8 T Cells with a Highly Altered TCR Vb Repertoire but with an Unmodified Phenotype

**DOI:** 10.1371/journal.pone.0021240

**Published:** 2011-06-27

**Authors:** Nicolas Degauque, Françoise Boeffard, Yohann Foucher, Caroline Ballet, Sophie Brouard, Jean-Paul Soulillou

**Affiliations:** 1 INSERM, UMR643, Nantes, France; 2 CHU de Nantes, ITUN, Nantes, France; 3 Université de Nantes, Faculté de Médecine, Nantes, France; 4 INSERM, EA 4275, Nantes, France; Institut Pasteur, France

## Abstract

CD8 T cell clonal expansions (TCE) have been observed in elderly, healthy individuals as well in old mice, and have been associated with the ageing process. Both chronic latent and non-persistent viral infections have been proposed to drive the development of distinct non-functional and functional TCE respectively. Biases in TCR Vβ repertoire diversity are also recurrently observed in patients that have undergone strong immune challenge, and are preferentially observed in the CD8 compartment. Healthy adults can also exhibit CD8 T cells with strong alterations of their CDR3 length distribution. Surprisingly, no specific investigations have been conducted to analyze the CD8 T cell repertoire in normal adults, to determine if such alterations in TCR Vβ repertoire share the features of TCE. In this study, we characterized the phenotype and function of the CD8 population in healthy individuals of 25–52 years of age. All but one of the EBV-positive HLA-B8 healthy volunteers that were studied were CMV-negative. Using a specific unsupervised statistical method, we identified Vβ families with altered CDR3 length distribution and increased TCR Vβ/HPRT transcript ratios in all individuals tested. The increase in TCR Vβ/HPRT transcript ratio was more frequently associated with an increase in the percentage of the corresponding Vβ^+^ T cells than with an absence of modification of their percentage. However, in contrast with the previously described TCE, these CD8^+^ T cells were not preferentially found in the memory CD8 subset, they exhibited normal effector functions (cytokine secretion and cytotoxic molecule expression) and they were not reactive to a pool of EBV/CMV/Flu virus peptides. Taken together, the combined analysis of transcripts and proteins of the TCR Vβ repertoire led to the identification of different types of CD8^+^ T cell clone expansion or contraction in healthy individuals, a situation that appears more complex than previously described in aged individuals.

## Introduction

Clonal CD8^+^ T cell expansion in healthy individuals has been reported as being associated with the ageing process [1], [2], [3]. Such expansions are frequently identified in the elderly (one third of adults over the age of 65 years develop CD8 clonal expansions) and in aged animals (e.g. mice >2 years of age) (reviewed in [4]). Among the typical features of clonal CD8^+^ T cell expansions (reviewed in [5]), these cells exhibit mainly a CD8^+^ memory T-cell phenotype based on the expression of cell surface markers such as CD45RA/CCR7, and respond poorly to TCR stimulation with low proliferation and cytokine production [6], [7], [8], [9]. Whereas these clones are not associated with overt diseases or malignancies [2], they may negatively impact the immune system by generating gaps in the TCR repertoire. Chronic antigen stimulation is thought to drive the expansion of clonal CD8^+^ T cells, as individuals with such clonal CD8^+^ T cell expansions frequently test positive for persistent virus such as CMV [8]. Conversely, clonal CD8^+^ T cell expansions can arise in the absence of persistent virus after the successful resolution of an acute infection [10], [11]. Interestingly, abundant CD8^+^ T cell clones are also observed in patients undergoing immune system stimulation, such as with allogeneic transplantation or chronic viral infection, and a less diverse TCR Vβ repertoire with strong alterations of the CDR3 length distribution is observed [12], [13].

In our previous reports, we have accumulated observations that peripheral CD8 repertoire alterations are not restricted to the elderly but can also be observed in apparently normal adults [12], [13], [14]. So far, such alterations have only been characterized for their altered CDR3 length distribution, and a detailed description and characterization of these clonal expansions is lacking.

In this report, we performed a detailed study of the CD8 TCR Vβ repertoire alterations as well as phenotype and function in healthy adult volunteers. The blood donors were specifically chosen as EBV-positive HLA-B8, as the necessary tools such as tetramers and known virus-derived peptides were available. Of note, only one out of 8 studied individuals was positive for CMV infection. Our data show that large changes in the CD8 TCR Vβ repertoire can be identified by spectratyping in healthy adult individuals. These CD8^+^ clonal expansions, which correspond to an increase in the percentage of Vβ_x_^+^ CD8^+^ T cells with a dominant single CDR3 length, are more frequent than CD8^+^ clonal restrictions (i.e. no modification in the percentage of Vβ_x_^+^ CD8^+^ T cells with a dominant single CDR3 length). However, despite the use of strongly selected CDR3 length distributions, the CD8^+^ T cells do not exhibit a unique phenotype but instead display a normal effector function (in terms of cytokine secretion and cytotoxic molecule expression). Finally, these CD8^+^ clonal expansions do not contain EBNA-3 specific T cells and are not reactive to a pool of peptides derived from EBV, CMV and Flu viruses. Taken together, CD8^+^ clonal expansions are commonly identified in healthy adults and may exist independently of chronic virus infection.

## Results

### “Unusual” usage of Vβ families within the CD8^+^ T cell compartment of healthy individuals

**Figure 1:**
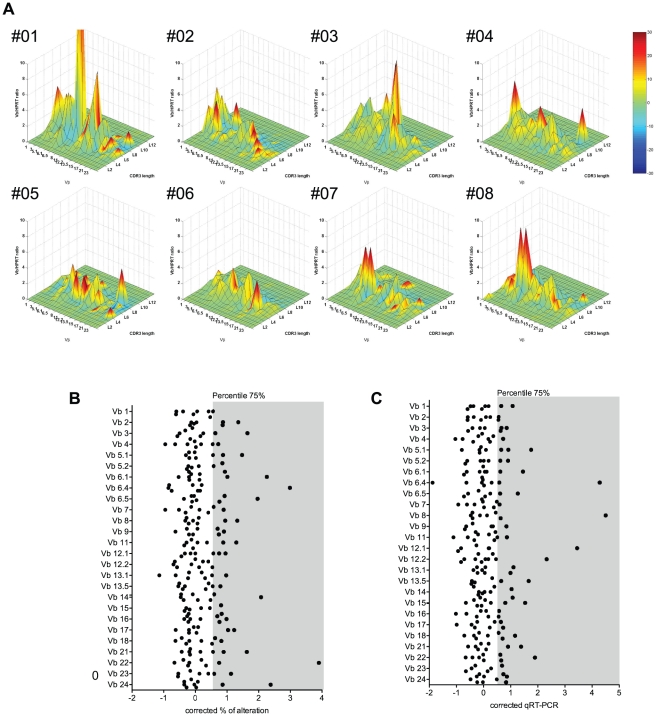
Identification of “unusual” transcriptional usage of Vβ families within the CD8^+^ T compartment of healthy individuals. (A) TCR Vβ repertoire analysis by TcLandscape of 8 healthy individuals (HLA-B8). The X-axis displays the 26 Vβ families analyzed, the Y-axis the CDR3 lengths, the Z-axis the Vβ/HPRT transcript ratio and the colors represent the percentage of alteration. (B) Identification of Vβ families with a corrected qRT-PCR value above the 75% percentile. Vβ transcripts were quantified by qRT-PCR. Each dot represents the individual corrected qRT-PCR value for a given Vβ family. The gray section represents the 75% percentile and defines the boundary of “unusual” usage of a Vβ family. (C) Identification of Vβ families with a corrected % of alteration value above the 75% percentile. The percentage of alteration for each Vβ family was evaluated as previously described [14], [15]. Each dot represents the individual corrected % of alteration value for a given Vβ family. The gray section represents the 75% percentile and defines the boundary of “unusual” usage of a Vβ family. See the statistical method within the Materials & Methods section for a detailed description of the statistical methodology.

The CD8 T cell TCR Vβ repertoire in 8 healthy adult individuals (median age 39 years, range 25–52 years) was analyzed using the TcLandscape technology (Figure 1) [15]. TcLandscape technology has proven to be a useful technic to globally assess TCR Vβ repertoire. For each of the 26 Vβ families (x-axis), the CDR3 length-distribution is measured (y-axis), and compared to a reference gaussian CDR3 length-distribution. The percentage of alteration of this Vβ_n_ CDR3 length-distribution is indicated by the red color code ranging from green color (i-e gaussian distribution) to red color (i-e highly altered CDR3 length distribution). Transcript amount of each Vβ families is shown using the z-axis. For each individual, some Vβ families exhibited an alteration in their CDR3 length distribution, highlighting clonal selections. One hurdle to the global assessment of the TCR Vβ repertoire is the identification of an “unusual” usage of specific Vβ families based on the % of alteration of the CDR3 length-distribution and the Vβ/HPRT transcript ratio. In order to overcome this problem, we developed an unsupervised statistical method combining a normalization of the values for each Vβ family with a global appraisal of the “usual” usage of Vβ families. This method takes into account the different range for the qualitative (percentage of alteration of the CDR3 length distribution) and the quantitative (Vβ/HPRT transcript ratio determined by qRT-PCR) parameters of the various Vβ families and the size of the analyzed population. The third quartile (i.e. percentile 75%) was calculated for the Vβ/HPRT transcript ratio (Figure 1.B) and for the % alteration of CDR3 length distribution (Figure 1.C). This analysis revealed a strong heterogeneity in the association between CDR3 length distribution alteration and the Vβ/HPRT transcript ratio. Indeed, only a limited number of Vβ families exhibited both an altered CDR3 length distribution and a high Vβ/HPRT transcript ratio (11% of Vβ families were above the third quartile; Table 1). Hereafter, these Vβ families will be referred to as “P-Vβ families” as these Vβ families can be visualized as red peaks in the TcLandscape view.

**Table 1:** Frequency of Vβ families with “unusual” usage identified at the transcriptional level.

Frequency of Vb outside the IQR_25–75%_				
% of alteration & qRT-PCR	% of alteration	qRT-PCR	% of alteration only	qRT-PCR only
11.54%	25%	25.48%	13.46%	13.94%

The percentage of Vβ families with a percentage of alteration of the CDR3 length distribution and/or a qRT-PCR value above the third quartile was evaluated with a specific unsupervised statistical method to identify “unusual” usage of each Vβ family.

### P-Vβ families are preferentially associated with an increase in percentage of Vβ^+^ CD8^+^ T cells

**Figure 2:**
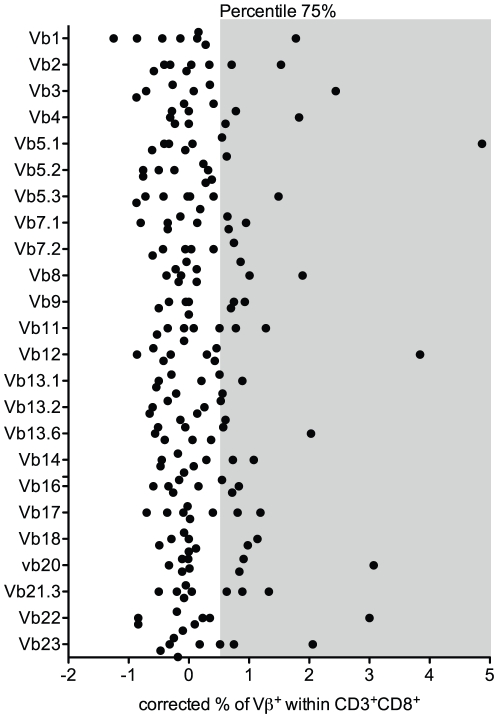
Identification of an “unusual” frequency of Vβ families within CD8^+^ T cells. Identification of Vβ families with a corrected percentage of Vβ^+^ within CD3^+^CD8^+^ cells above the 75% percentile. The frequency of Vβ^+^ cells within the CD3^+^CD8^+^ cells was corrected using the methodology described in the statistical part of the Materials & Methods section. The gray section represents the 75% percentile and defines the boundary of “unusual” usage of a Vβ family.

Based on the literature, it is assumed that selection of a Vβ_x_ family identified by an alteration of the CDR3 length distribution is also associated with an increase in the percentage of Vβ_x_^+^ T cells characterized by flow cytometry [16]. To investigate whether the Vβ families identified on the basis of their transcription were indeed also associated with an increase in the corresponding Vβ^+^ T cells, we characterized the frequency of each Vβ family within the CD3^+^CD8^+^ compartment. Using the same unsupervised statistical method, P-Vβ_x_ families with a percentage of Vβ_x_^+^ T cells above the third quartile were identified (Figure 2).

We restricted our analysis to the 14 Vβ families for which anti-Vβ mAb and Vβ specific primers were available (by flow cytometry and TcLandscape respectively; Table S2). Among the P-Vβ families, 71% were indeed found to be over-represented in terms of number of cells corresponding to the P-Vβ families (Table 2).

**Table 2:** Identification of Vβ families with “unusual” usage identified at the transcriptional level and at the protein level.

mRNA or protein level	Parameter analyzed to identify “unusual” Vβ families	Individuals id	Vβ families name				
protein	% of TCR Vβ within CD8^+^ T cells	#01	Vβ1	**Vβ2**	Vβ4	Vβ5.1	**Vβ8**
		#02	**Vβ2**	**Vβ5.1**	Vβ17		
		#03	Vβ5.1	Vβ8	**Vβ14**	Vβ16	
		#04	**Vβ3**	Vβ4	**Vβ11**	Vβ16	Vβ18
		#05	Vβ4	Vβ9	Vβ18	**Vβ22**	Vβ23
		#06	Vβ9	**Vβ17**			
		#07	Vβ14	**Vβ16**	Vβ23		
		#08	Vβ9	Vβ11			
mRNA	% of alteration & Vβ/HPRT ratio	#01	**Vβ2**	**Vβ8**	Vβ18	Vβ23	
		#02	**Vβ2**	**Vβ5.1**			
		#03	Vβ4	**Vβ14**			
		#04	**Vβ3**	**Vβ11**			
		#05	**Vβ22**				
		#06	**Vβ17**				
		#07	**Vβ16**				
		#08	Vβ23				

The same specific unsupervised statistical method was used to identify “unusual” usage of each Vβ family at both the transcriptional and protein level. Vβ families with an “unusual” usage at both the transcriptional and protein level are highlighted in bold.

Based on the variation of the number of the corresponding Vβ^+^ T cells, we thus identified two types of P-Vβ families. Type 1 (referred to as clonal expansion), characterized by an increase in the frequency of P-Vβ family T cells, was the most commonly observed and represented 10 out of 14 P-Vβ families. Type 2 (referred to as clonal restriction), characterized by a normal frequency of P-Vβ family T cells, was less frequent (4 out of the 14 P-Vβ families studied).

### Intra and extracellular density of TCR Vβ proteins on P-Vβ^+^ family T cells

**Figure 3:**
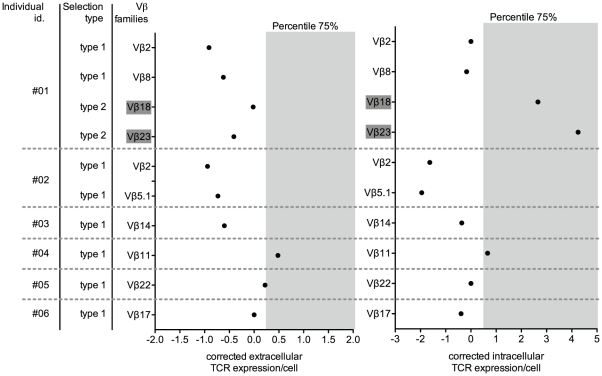
Intracellular and extracellular density of TCR Vβ expression by CD8^+^ T cells with P-Vβ families. Cell surface and intracellular TCR Vβ expression at the cell surface of CD3^+^CD8^+^ T cells was quantified per cell using QuantiBRITE™ PE beads. The quantification of TCR Vβ expression per cell was performed for Vβ families with a type 1 selection (i.e. clonal expansion) or a type 2 selection (i.e. clonal restriction; dark gray). Extracellular and intracellular TCR Vβ expression per cell was corrected using the methodology described in the statistical part of the Materials & Methods section. The gray section represents the 75% percentile and defines the boundary of “unusual” usage of a Vβ family.

The presence of a high level of Vβ transcripts is usually associated with an increase in the frequency of the corresponding Vβ^+^ CD8^+^ T cells. However, an increase in Vβ protein expression per cell could be associated with a high level of Vβ transcripts without any increase in the percentage of Vβ^+^ CD8^+^ T cells. To test this hypothesis, the number of TCR Vβ proteins was evaluated at the surface and in the intracellular compartment of CD8^+^ T cells by combining anti-TCR Vβ mAb staining and the use of Quantibright Beads (BD Biosciences). We observed that only 25% of P-Vβ families with a type 1 selection (i.e. clonal expansion) exhibited an increase in TCR Vβ protein at the cell surface (Vβ11 ind.#04 and Vβ22 ind.#05; Figure 3). No modification of the intracellular TCR Vβ protein expression was observed for the P-Vβ families with a type 1 selection. In contrast, P-Vβ families with a type 2 selection (i.e. clonal restriction) did not express more TCR Vβ protein at the cell surface but displayed increased intracellular TCR Vβ protein expression (Vβ18 and Vβ23 ind.#01; Figure 3).

### CD8^+^ T cells with and without P-Vβ families exhibit a similar phenotype

**Figure 4:**
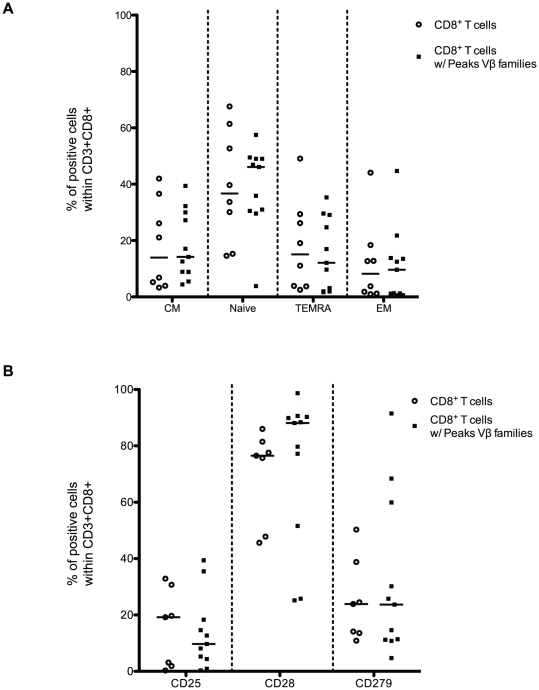
CD8^+^ T cells with and without P-Vβ families exhibit a similar phenotype. Expression of CCR7 and CD45RA (A) or CD25, CD28 and CD279 (B) by CD8^+^ T cells and by CD8^+^ T cells with P-Vβ families was measured by flow cytometry. Based on the expression of CCR7 and CD45RA, four populations were measured: naive cells (CD45RA^+^CCR7^+^), central memory (CM) cells (CD45RA^−^CCR7^+^), effector memory (EM) cells (CD45RA^−^CCR7^−^), and effector (EMRA) cells (CD45RA^+^CCR7^−^). The percentage of positive cells within CD3^+^CD8^+^ cells or CD8^+^ T cells with P-Vβ families is shown.

Because clonal CD8^+^ T cell expansions exhibit mainly a memory T-cell phenotype [17], [18], we characterized the phenotype of the “Peak Vβ families”. On the basis of CD45RA and CCR7 expression, four populations of CD8 T cells were classified as naïve cells (CD45RA^+^CCR7^+^), central memory (CM) cells (CD45RA^−^CCR7^+^), effector memory (EM) cells (CD45RA^−^CCR7^−^) and effector (EMRA) cells (CD45RA^+^CCR7^−^) [19]. No difference in the frequency of the 4 sub-populations was found between CD8^+^ T cells with P-Vβ families and the whole CD8^+^ T cells (Figure 4.A).

Down-regulation of CD28 is generally associated with CD8^+^ T cell differentiation, whereas up-regulation of CD25 is associated with an activated state. The inhibitory molecule CD279 is up-regulated by exhausted CD8^+^ T cells. We further characterized the phenotype of these CD8^+^ T cells and we found that CD8^+^ T cells with P-Vβ families exhibited weak expression of CD25 (median 9.7 vs. 19.2 between CD8^+^ T cells with P-Vβ families and whole CD8^+^ T cells respectively) and a similar high level of CD28 (median 88.1 vs. 76.5 between CD8^+^ T cells with P-Vβ families and whole CD8^+^ T cells respectively) (Figure 4.B). Taken together, CD8^+^ T cells with P-Vβ families did not preferentially exhibit a unique phenotype.

### CD8^+^ T cells with P-Vβ families secrete cytokines upon polyclonal stimulation

**Figure 5:**
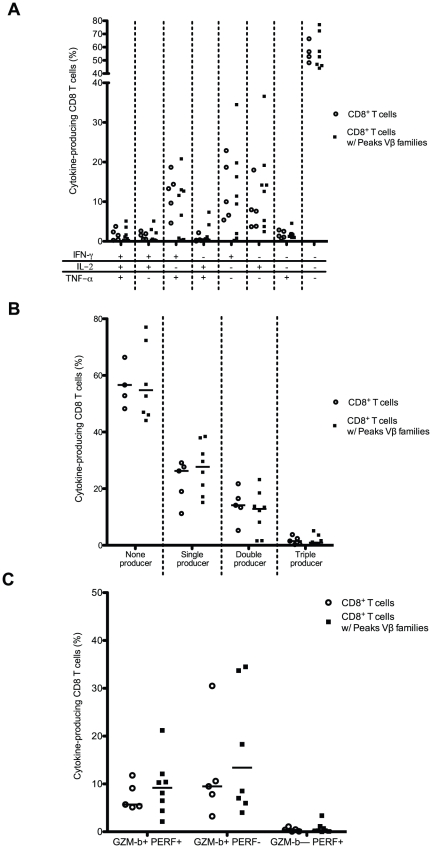
Cytokine and cytotoxic molecule profile of CD8^+^ T cells with P-Vβ families. (A) Functional characterization of IFN-γ, TNF-α and IL-2 by CD8^+^ T cells and CD8^+^ T cells with P-Vβ families. Purified T cells were stimulated for 6 hours with PMA and ionomycin and then analyzed by seven-color flow cytometry. Boolean gating was done to separate the 7 distinct populations based on the production of IFN-γ, TNF-α and IL-2 in any combination. (B) Frequency of the various cytokine producers (none to three cytokine producers) in CD8^+^ T cells and CD8^+^ T cells with P-Vβ families. (C) Expression of GZM-B and PERF by CD3^+^CD8^+^ T cells and CD8^+^ T cells with P-Vβ families.

A weak response of clonal CD8^+^ T cell expansions to polyclonal stimulation has been described [6], [7], [8], [9]. To look at the function of CD8^+^ T cells with P-Vβ families, cytokine production and cytotoxic molecule expression were analyzed. CD8^+^ T cells were first stimulated with PMA and ionomycin for 6 hours and production of IFN-γ, TNF-α and IL-2 was analyzed (Figure 5.A and B). The frequency of cells secreting at least one cytokine was similar for CD8^+^ T cells with P-Vβ families and for the whole CD8^+^ T cell population (40.6%±5.8 vs. 38.6%±6.3 respectively). The quality of the CD8 response (i.e. the ability to secrete more than one cytokine) was then analyzed. The frequencies of single, double or triple-cytokine producers, as well as the nature of the cytokine secreted, were also comparable for CD8^+^ T cells with P-Vβ families and for the whole CD8^+^ T cell population (Figure 5.A and B). No difference in the expression of cytotoxic molecules (GZM-B and PERF) was observed between CD8^+^ T cells with P-Vβ families and the whole CD8^+^ T cell population (Figure 5.C). Collectively, these data obtained after 6 hours of stimulation, an optimal timing for the analysis of antigen-experienced T cell responses, suggest that CD8^+^ T cells with P-Vβ families do not exhibit the effector function characteristics of memory cells, including the expression of cytotoxic molecules and the ability to secrete multiple cytokines, and primarily behave as normal CD8^+^ T cells.

### P-Vβ families are distinct from the Vβ families of EBNA-3A-specific CD8^+^ T cells

**Figure 6:**
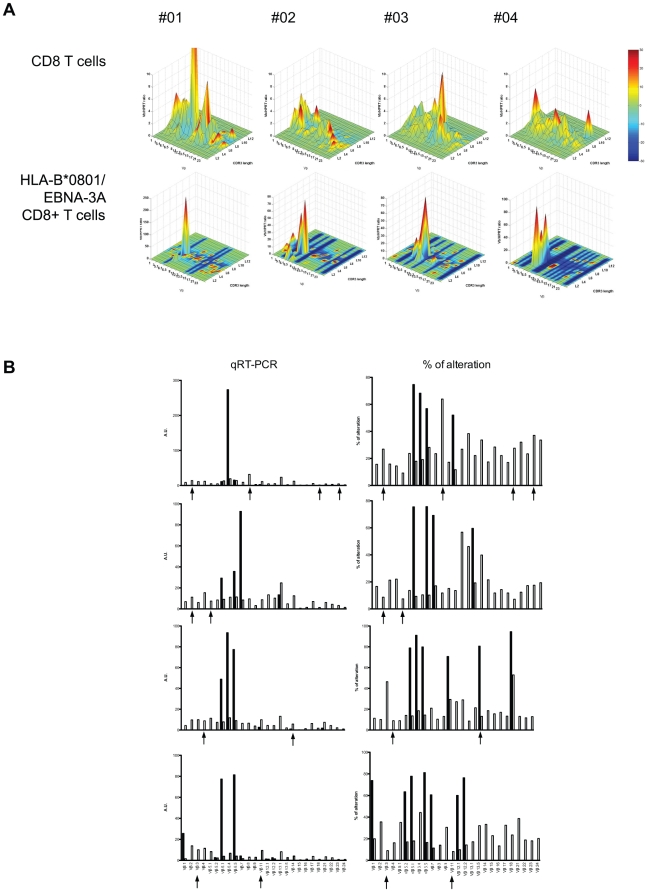
CD8^+^ T cells with P-Vβ families are distinct from EBNA3 latent epitope- specific Vβ families. CD8^+^ T cells (white bar) and HLA-B*0801/EBNA-3A CD8^+^ T cells (black bar) were FACS-sorted from 4 HLA-B8 healthy individuals (HV #01 to #04). TCR Vβ repertoire usage was characterized by qRT-PCR (left column; arbitrary unit) and by spectratyping (right column; % of alteration). Black arrows indicate the P-Vβ families previously identified.

Because chronic immune responses against herpes virus can shape the CD8 repertoire [8], [20] and that HLA-B8 individuals have been shown to develop public anti-EBV responses [21] that can be studied by HLA-B*0801/EBNA-3A tetramers, we tested whether the observed TCR Vβ repertoire alteration could be explained by EBV infection. An HLA-B*0801/EBNA-3A (FLRGRAYGL) pentamer was used to isolate EBV-specific cells. FLRGRAYGL peptides derived from the latent cycle antigen EBNA-3A is among the strongest EBV latent cycle epitopes [22], [23]. TCR Vβ repertoire usage was compared between CD8^+^ T cells and HLA-B*0801/EBNA-3A CD8^+^ T cells (Figure 6). Highly restricted TCR Vβ repertoires were observed in the HLA-B*0801/EBNA-3A CD8^+^ T cell fraction, characterized by the use of a limited number of Vβ families and a high Vβ/HPRT transcript ratio. A recurrent peak within the Vβ6 family was observed in all patients (Figure 6), using a similar CDR3 length distribution (3 out of 4 patients exhibited a similar CDR3 length for the Vβ6.1 family and 4 out of 4 for the Vβ6.5 family; Figure S1). TCR sequencing of the Vβ6.1 and 6.5 family PCR products identified two TCR sequences, sharing the TRBJ2-7 gene and two CDR3 sequences (AGC TT/CA or TCA GGA CAG GCC), similar to the public selection previously reported (6S8 gene analysis is shared between Vβ6.1 and Vβ6.5 families when these families are analyzed by TcLandscape; Table S2) [24]. Of interest, none of the P-Vβ families were found in the HLA-B*0801/EBNA-3A CD3^+^CD8^+^ cell fraction (Figure 6).

## Discussion

Significant changes in the blood CD8 T cell TCR Vβ repertoire have been reported in normal mice and humans, and are usually associated with ageing of the immune system [2], [6], [25]. In some cases, CD8 Vβ clonal expansions have been shown to occupy up to 50% of the total CD8Vβ T cell repertoire in the elderly [2] and up to 80% of the total CD8Vβ T cell repertoire in old mice [6]. It has been proposed that infection with persistent or transient viruses could induce the expansion of CD8 V β clones [8], [10], [11]. We now report that healthy adult individuals also commonly exhibit alterations of the TCR Vβ repertoire within the CD8 compartment, as identified by spectratyping. These P-Vβ families are mainly associated with an increase in the corresponding percentage of Vβ^+^ T cells. Despite the use of a highly selected CDR3 length distribution, these CD8^+^ T cells do not exhibit a bias in their phenotype and maintain normal effector function after short-term polyclonal stimulation (in terms of cytokine secretion and expression of cytotoxic molecules).

The ability to identify and to study CD8^+^ clonal expansions relies on the method used. CD8^+^ clonal expansions can be identified by measuring the size of the TCR Vβ_x_ CD8^+^ T cell population (using anti-Vβ mAb) or by characterizing the magnitude of skewing of CDR3 length distribution. Most studies identify CD8^+^ clonal expansions when the percentage of a given TCR Vβ CD8^+^ T cell in elderly individuals is 2 to 3 standard deviations above the mean of this TCR Vβ in young individuals [2], [6]. Spectratyping is used to confirm the clonality of the TCR within the CD8^+^ clonal expansion. However, the number of individuals included often precludes the use of criteria based on parametric statistics. Moreover, we observed that the distribution of the % of alteration of CDR3 length distribution and the Vβ/HPRT ratio was heterogeneous for some Vβ families, suggesting a lack of Gaussian distribution in the usage of Vβ families in blood T cells of healthy adults. Thus, we first developed an unsupervised and non-parametric methodology to undertake a more precise characterization of the T cell repertoire in healthy individuals. Spectratyping (to analyze CDR3 length distribution) and qRT-PCR (to measure Vβ/HPRT ratio) were used to characterize the TCR Vβ repertoire. Various analyses based on the use of flow cytometry can be used to measure the frequency of CD8^+^ T cells expressing a given TCR Vβ as well as the expression of TCR Vβ per cell, either at the cell surface or in the cytoplasm. This non-parametric analysis was designed to take into account the number of individuals included, the variability of usage between the different Vβ families and the absence of normality in the usage of Vβ families. Using this approach, we identified various usages of TCR Vβ transcripts based either on the quantity of Vβ/HPRT transcript ratio or the alteration of CDR3 length distribution. Modification of CDR3 length distribution was associated with an accumulation of transcripts in only half of cases.

**Figure 7:**
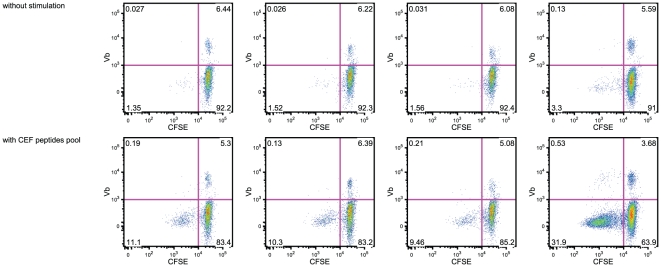
Stimulation by CMV/EBV/FLU peptides does not elicit the proliferation of CD8^+^ T cells with P-Vβ families. CFSE labeled PBL were cultured for 6 days in the presence of CMV/EBV/FLU peptide cocktail (CEF peptide pool 2.5 ug/mL) and anti-CD49d and anti-CD28.2 mAb (2 ug/mL). On day 3, 1 mL of fresh media was added. On day 6, the CFSE dilution was analyzed by gating on live CD3^+^CD8^+^ cells and live CD3^+^CD8^+^ Vβ^+^ cells.

The characteristics of the CD8^+^ T cells we observed in our adult population are rather different from those published in aged populations. The average age of the healthy volunteers in this study was 39 years (age range 25–52 years; Table S1). Among the differences in their characteristics, the clonal CD8^+^ T cells exhibited a distribution in the various phenotype (naive, CM, EM and TEMRA cells), which was similar to that of the whole CD8^+^ T cell population. In contrast, previously published clonal CD8^+^ T cell expansions were mainly found to exhibit a memory phenotype. The phenotype of clonal CD8+ T cell expansions thus remained an open question. The origin of clonal CD8+ T cell expansions has not been identified and it is assume to be driven by various mechanisms. It is likely that memory CD8 T cell and clonal CD8+ T cell expansions represent two different entities. For instance, the memory compartment contributes to less than 1 percent of the total diversity of the TCR ab repertoire [26] and efficiently responds to a second antigeneic challenge. In contrast, the response to TCR stimulation to CD8 clonal expansion is diverse, ranging from an absence of response [7], [11] to an efficient cytokine secretion (figure 5 and [7]).

The TCR reactivity of the CD8 clones found in our adult cohort remains to be elucidated, as for the clonal expansions of CD8^+^ T cells found in the elderly. As persistent viruses such as CMV or EBV are known to impact the CD8 repertoire, we investigated a possible reactivity for viral peptide of the CD8^+^ T cells with clonal expansions. It is important to note that whereas all 8 healthy individuals had tested positive for EBV, only one was positive for prior CMV infection. Thus, in our study, we excluded the possibility of CMV infection imprinting the TCR Vβ repertoire. These clonal CD8^+^ T cells were not found in the HLA-B*0801/EBNA-3A CD3^+^CD8^+^ cell fraction (Figure 6). Moreover, stimulation with a pool of CMV, EBV and Flu peptides (CEF Class I Peptide Pool) did not elicit strong IFN-γ production by clonal CD8^+^ T cells (Figure 7). Although this virus peptide pool triggers proliferation of the whole PBL population, it is noticeable that none of the P-Vβ families studied was responsive. In addition, no advantage or disadvantage was found for the clonal expansions of CD8^+^ T cells in terms of cytokine response or expression of cytotoxic molecules upon polyclonal stimulation (Figure 5). Our data suggest that the CD8^+^ clones are not directly related to the presence of persistent pathogens, as for previous studies on mouse CD8^+^ clonal expansions which occur in the majority of mice by 2 years of age even in a specific pathogen-free environment [3], [6], [27]. The distribution of CD8^+^ clonal expansions is random with a broad variety of TCR Vβ chains detected in different CD8^+^ expansions even in a colony of genetically identical mice. It has to be noted that CD8^+^ clonal expansions have also been identified in mouse models in which viral agent had been successfully cleared by the immune system [11]. CD8^+^ clonal expansions induced by non-persistent antigen have been reported to retain effector functions [10]. Altogether, these observations suggest that, in healthy individuals, CD8 T cell clones escape post-infection homeostatic contraction. The specificity of these clones remains to be determined but it is likely that their specificity is towards non-persistent viruses.

In conclusion, we show that the CD8 repertoire of normal adult humans is not Gaussian. The combined analysis of transcripts and proteins of the TCR Vβ repertoire enabled the identification of different types of CD8^+^ T cell clone expansions or contractions in healthy individuals, a situation that appears more complex than previously described in aged individuals. Notably, these CD8^+^ T cell clones exhibit a diverse phenotype and differ from clonal CD8^+^ T cell expansions observed in the elderly in that they exhibit a normal response to polyclonal stimuli.

## Materials and Methods

### Subjects and Ethics statement

Peripheral blood lymphocytes (PBL) were collected from 8 HLA-B8^+^ EBV^+^ healthy volunteers, enrolled by the Etablissement Français du Sang (EFS, Nantes, France) within the context of a research contract. All donors were informed of the final use of their blood and signed an informed consent. The approval of an ethical committee was thus not necessary. The demographic characteristics of the healthy volunteers are presented in Table S1. All patients were EBV^+^ but only 1 out of 8 tested positive for CMV.

### Cell isolation

PBL were separated by Ficoll density centrifugation (LMS Eurobio) or were enriched in CD3 cells by elutriation (DTC core-facility IFR 26, Nantes). Untouched CD8^+^ T cells were purified using the CD8^+^ T cell isolation kit (Miltenyi) according to the manufacture's recommendations. To isolate HLA-B*0801/EBNA-3A^+^ T cells (referred to as HLA-B*0801/EBNA-3A CD8^+^ T cells), PBMC were stained with CD3-APC, CD8-FITC and PE-labeled HLA-B*0801/FLRGRAYGL (EBV EBNA-3A) Pro5™ MHC Pentamer (ProImmune). HLA-B8 EBV^+^ CD8^+^ T cells were then isolated using a high-speed cell sorter (FACSAria; BD Biosciences). Purity was greater than 98%.

### Characterization of the TCR Vβ repertoire

#### Transcription

RNA was extracted from purified CD8^+^ T cells or HLA-B8 EBV^+^ CD8^+^ T cells using NucleoSpin RNA II (Macherey-Nagel) according to the manufacture's procedure. Total RNA was reverse-transcribed using an Invitrogen cDNA synthesis kit (Boehringer Mannheim). The TcLandscape® was performed as previously described by combining the CDR3 length distribution (CDR3-LD) with each normalized amount of Vβ transcript [14], [15]. Briefly, CDR3-LD was determined by amplifying the cDNA by PCR using pairs of primers specific for each Vβ gene [12] followed by length separation using a capillary sequencer (Applied Biosystems 3730) [28]. In parallel, the level of Vβ family transcripts was measured by qRT-PCR and normalized by a housekeeping gene (HPRT).

#### Vβ frequency analysis

PBMC were stained with Alexa 700-conjugated anti-CD3, Pacific-Blue-conjugated anti-CD8 antibodies and the various anti-Vβ family antibodies included in the IOTest® Beta Mark (PN IM3497; Beckman Coulter) according to the manufacture's guidelines. The frequency of the 24 Vβ families was evaluated by gating on CD3^+^CD8^+^ T cells with FlowJo Software (TreeStar).

#### Estimation of the TCR Vβ expression per cell

The number of TCR Vβ proteins expressed per CD8^+^ T cell was assessed using the QuantiBRITE™ PE beads (BD Biosciences) according to the manufacturer's guidelines. For each anti-TCR Vβ-specific mAb, the Fluorochrome/Protein ratio was obtained from the antibody provider (Beckman Coulter).

### Flow cytometry

CD8^+^ T cells and specific Vβ_n_ CD8^+^ T cells were analyzed by multi-color flow cytometry. Samples were stained with labeled antibodies directed against cell surface markers for 30 minutes at 4°C. Antibodies raised against the following antigens were used to characterize the memory/activated phenotype of the CD8^+^ T cells: FITC-conjugated CD28; Alexa 700-conjugated CD3; Pacific-Orange-conjugated CD8; PE-Cy7-conjugated CD197 (CCR7); PE-Cy5-conjugated CD45RA; Alexa 647-conjugated CD279; and PE-conjugated specific anti-Vβ. All antibodies were purchased from BD Biosciences except for the anti-Vβ antibodies which were purchased from Beckman Coulter. To detect intracellular proteins, samples were first labeled with LIVE/DEAD Fixable Aqua stain according to the manufacturer's guidelines, and then labeled with PE-Cy7-conjugated anti-CD3 mAb, Alexa700-conjugated anti-CD8 mAb and the various PE-conjugated anti-Vβ mAb. The samples were then permeabilized and fixed with Perm/Fix reagent (eBiosciences), and finally stained for 30 min at 4°C with APC-conjugated anti-IFN-γ mAb, V450-conjugated anti-IL-2 mAb and FITC-conjugated anti-TNF-α mAb. Samples were acquired on a BD LSR II BD Biosciences and analyzed with FlowJo Software (TreeStar).

### Intracellular cytokine production

#### Mitogenic stimulation

1×10e6 T cells were cultured in 96-U bottom plates for 6 hours in complete medium in the presence of PMA (20 ng/mL) and Ionomycin (400 ng/mL). After 1 hour, BFA (10 ng/mL) was added. Samples were stained as described in the “Flow cytometry” section to detect IFN-γ, TNF-α and IL-2 secretion.

### Detection of cytokine-secreting CD8^+^ T cells following stimulation with viral peptides

Purified PBMC were thawed, resuspended in complete medium (+10 U/mL DNase I) at a final concentration of 2–4×10e6/mL and cultured overnight. Cells were stained with CFSE according to the manufacturer's recommendations and plated at 4×10e6 cells/well in complete medium with CEF Class I Peptide Pool “Plus” (2.5 µg/mL; Cellular Technology Ltd.), anti CD28.2 mAb and anti-CD49d mAb (2 µg/mL) for 6 days. CEF Class I Peptide Pool “Plus” covers HLA class I restricted T cell epitopes of CMV, EBV and Flu virus. On day3, 1 mL of fresh medium was added. BFA (10 ng/mL) was added for the last 5 hours of stimulation. Samples were stained as described in the “Flow cytometry” section.

### Statistical methods

There is no gold standard to identify the abnormal % of alteration or Vβ_n_/HPRT ratio. Most studies identify CD8^+^ clonal expansions when the percentage of a given TCR Vβ CD8^+^ T cells in aged individuals is 2 to 3 standard deviations above the mean of this TCR Vβ in young individuals [2], [6]. However, the methodology used in previous studies assumes the normality in the usage of Vβ families, a large number of individuals and does not take into account the variability of usage between the different Vβ_n_ families. Of note, we observed that the distribution shape of the % of alteration and the Vβ_n_/HPRT ratio were heterogeneous for some Vβ_n_ families, suggesting a lack of Gaussian distribution. Thus, we proposed a unsupervised and non-parametric methodology, specifically devoted to the identification of “unusual” usage of Vβ families within the TCR Vβ repertoire For each parameter measured for each Vβ family, the 8 individual values were transformed by subtracting the median and by dividing by the InterQuartile Range (IQR). When all the transformed values regardless the Vβ family were plotted together, a skewed distribution was observed with a threshold at the 75% percentile, that identified the unusual values. No assumption was associated with this method, which is completely non parametric and descriptive. It would not have been possible to propose statistical tests, especially regarding the heterogeneity of the distributions and the high number of parameters in comparison to the number of individuals. All the statistical analysis were performed using R software [29] and the figures were done using Graphpad Prism.

## Supporting Information

Figure S1 HLA-B*0801/EBNA-3A CD8^+^ public clones. HLA-B*0801/EBNA-3A CD8^+^ T cells from 4 HLA-B8 healthy volunteers (HV #01 to #04) were FACS-sorted and the TCR Vβ repertoire was analyzed by TcLandscape. (A) Spectratyping of Vβ families 6.1 and 6.5 show the usage of similar CDR3 lengths across the individuals. (B) Identification of two public sequences within PCR products of Vβ families 6.1 and 6.5.
(EPS)

Table S1 **Demographic characteristics of the enrolled healthy volunteers.**
(DOCX)

Table S2 **Vβ family genes analyzed using the TcLandscape technique or anti-Vβ mAb.** The Vβ family names indicated on the TcLandscape refer to the Arden nomenclature. The IMGT nomenclature is mentioned for the Vβ families that are identical between the two techniques.
(DOCX)
