# Supplementary material for: The Blood of Healthy Individuals Exhibits CD8 T Cells with a Highly Altered TCR Vb Repertoire but with an Unmodified Phenotype
Source: PLoS One. 2011 Jun 27;6(6):e21240. doi: 10.1371/journal.pone.0021240 (PMC3124488; doi:10.1371/journal.pone.0021240)
Supplement: Table S1 — Demographic characteristics of the enrolled healthy volunteers. (DOCX) [file pone.0021240.s002.docx]

| **HV Id** | **HV gender** | **HV age (years)** | **EBV status** | **CMV status** |
| --- | --- | --- | --- | --- |
| #01 | M | 35 | + | - |
| #02 | M | 40 | + | - |
| #03 | F | 48 | + | - |
| #04 | M | 25 | + | - |
| #05 | F | 47 | + | + |
| #06 | F | 38 | + | - |
| #07 | M | 52 | + | - |
| #08 | F | 27 | + | - |
