# Supplementary material for: The Blood of Healthy Individuals Exhibits CD8 T Cells with a Highly Altered TCR Vb Repertoire but with an Unmodified Phenotype
Source: PLoS One. 2011 Jun 27;6(6):e21240. doi: 10.1371/journal.pone.0021240 (PMC3124488; doi:10.1371/journal.pone.0021240)
Supplement: Table S2 — Vβ family genes analyzed using the TcLandscape technique or anti-Vβ mAb. The Vβ family names indicated on the TcLandscape refer to the Arden nomenclature. The IMGT nomenclature is mentioned for the Vβ families that are identical between the two techniques. (DOCX) [file pone.0021240.s003.docx]

|  | **TcLandscape Vβ families** | **Analyzed genes** | **Anti-Vβ families mAb** | **Analyzed genes** | **IMGT nomenclature** |
| --- | --- | --- | --- | --- | --- |
| **Identical Vβ genes analyzed** | Vβ1 | 1S1 | Vβ1 | 1S1 | TRBV 9 |
|  | Vβ2 | 2S1 | Vβ2 | 2S1 | TRBV20-1 |
|  | Vβ3 | 3S1 | Vβ3 | 3S1 | TRBV 28 |
|  | Vβ4 | 4S1 | Vβ4 | 4S1 | TRBV 29-1 |
|  | Vβ5.1 | 5S1 | Vβ5.1 | 5S1 | TRBV 5-1 |
|  | Vβ8 | 8S1, 8S2, 8S3 | Vβ8 | 8S1, 8S2 | TRBV 12-3 / TRBV 12-4 |
|  | Vβ9 | 9S1 | Vβ9 | 9S1 | TRBV 3-1 |
|  | Vβ11 | 11S1 | Vβ11 | 11S1 | TRBV 25-1 |
|  | Vβ14 | 14S1 | Vβ14 | 14S1 | TRBV 27 |
|  | Vβ16 | 16S1 | Vβ16 | 16S1 | TRBV 12-5 |
|  | Vβ17 | 17S1 | Vβ17 | 17S1 | TRBV 19 |
|  | Vβ18 | 18S1 | Vβ18 | 18S1 | TRBV 18 |
|  | Vβ22 | 22S1 | Vβ22 | 22S1 | TRBV 2 |
|  | Vβ23 | 23S1 | Vβ23 | 23S1 | TRBV 13 |
| **Different Vβ genes analyzed** | Vβ5.2 | 5S2, 5S3, 5S4, 5S6 | Vβ5.2 | 5S2 |  |
|  | Vβ6.1 | 6S1, 6S2, 6S3, 6S6, 6S8 | Vβ5.3 | 5S3 |  |
|  | Vβ6.4 | 6S4 | Vβ7.1 | 7S1 |  |
|  | Vβ6.5 | 6S5, 6S8 | Vβ7.2 | 7S2 |  |
|  | Vβ7 | 7S1, 7S2, 7S3 | Vβ12 | 12S2 |  |
|  | Vβ12.1 | 12S1 | Vβ13.1 | 13S1 |  |
|  | Vβ12.2 | 12S2, 12S3 | Vβ13.2 | 13S2 |  |
|  | Vβ13.1 | 13S1, 13S2, 13S3, 13S6, 13S9 | Vβ13.6 | 13S6 |  |
|  | Vβ13.5 | 13S5 | Vβ20 | 20S1 |  |
|  | Vβ15 | 15S1 | Vβ21.3 | 21S3 |  |
|  | Vβ21 | 21S1, 21S2, 21S3 |  |  |  |
|  | Vβ24 | 24S1 |  |  |  |
